# Supplementary material for: Changes in Homogalacturonan Metabolism in Banana Peel during Fruit Development and Ripening
Source: Int J Mol Sci. 2021 Dec 27;23(1):243. doi: 10.3390/ijms23010243 (PMC8745247; doi:10.3390/ijms23010243)
Supplement: Supplementary file 1 [file ijms-23-00243-s001.zip › ijms-1380691-supplementary.pdf]

Supplementary data

Changes in Homogalacturonan Metabolism in Banana Peel during Fruit Development and Ripening

Tong Ning [1,†], Chengjie Chen [1,†], Ganjun Yi [2], Houbin Chen [1,3], Yudi Liu [1], Yanjie Fan [1], Jing Liu [1], Shule Chen [1], Sixuan Wei [1], Zexuan Li [1], Yehuan Tan [1], Zhenting He [1], and Chunxiang Xu [1,*]

†These authors contributed equally to this work.

*Author for correspondence: Chunxiang Xu

College of Horticulture

South China Agricultural University

483, Wushan Rd.

Guangzhou, 510642

Guangdong Province, China

Email: chxxu@scau.edu.cn, 970575851@qq.com

Table S1-2

Figure S1-2

Table S1. Primer sequences used in the present study.

Gene	Forward primer (5′-3′)	Reverse primer (5′-3′)
Actin	TGGTATGGAAGCCGCTGGTA	TCTGCTGGAATGTGCTGAGG
Ma09_g15970	ACTCTGTTCCCTGCACCAAC	GAGGTAAGCCCTCCGACAAG
Ma07_g28300	TCCTGAAGGGTGGAATGACTG	GCCATCAATGTATCCCCTGTC
Ma11_g23340	TTATTGACTTGGAGAAGTGGAG	GTAATGCAATGATGTAGCAGTC
Ma08_g12630	ACGGATGAAAGTGAGTGGAAG	CGCATCAGAAGTGATGGAG
Ma09_g12060	ACGCAATCGAGTTCAACATAAC	AGAGGCACTTGTTGTGGAAG
Ma03_g00210	TGCAAGCTTGTGGAAGAATTG	TCAGAAGTCAATGTGCCAACC
Ma04_g02960	CCAGTTCACTTGGCGTGTAGC	AAGCAGGTGATGGGAGGAATG
Ma07_g15530	CCTTGGACACACTGTACTACGC	GGTCAACCCAGCTGTGTACTTC
Ma03_g05670	GCAACGTGATAGTGAAGACC	TCCCCATAATACAACGTGTC
Ma02_g21670	AACTCTACTCCAGGGTGGTG	GAGGAGCCGTAGATGAACTG
Ma11_g02770	GAGGTCGAAGAAGCCGATATG	CCCAACACAATCCACAGGTAG
Ma02_g16140	CCAGTTCTACTGCGACCAGAG	TAGCTAGCTCCACGTTGCTTC
Ma05_g20750	TGAGAGGCAGGACACATGG	GACGATGTGATGGCAGGTG
Ma06_g30000	GCGAGTGGAAGAAGTGGAAC	CCCTTCTTGCACGAGAGAAC
Ma11_g19010	GATCGAGCACCGATGGAATC	AGAGGTTGTCGAGCGGACAC
Ma07_g18890	GGGTGCTCTGGAAACTTGG	CCCGGACATAAGGATGGTC
Ma08_g21130	ACGGCATCACCATCAAGAAC	CGGTGGAGCAGTATCCTGAG
Ma01_g07260	TTTTGGAGGACACAGGGTTC	TGCACTTGTACTGCCCGTAG
Ma04_g33470	ACCAGACGGCGTTCTTTGG	AAGGATCGGAAGGGCCAAG
Ma04_g28370	GAAGAAAACAACTCCAAGATGG	TGTGGGATATTCTAGTCCTTGC
Ma04_g23990	GACATCCAGACGTGGGTGAG	GCAGAGTTGAGCACGTTGATG
Ma04_g20800	ACCTCAGCAACGTCCAGACC	CCGAGTTGATTCACGAGAGC
Ma04_g31200	GACATGCAGACGTGGGTGAG	ATGACAAGGCGTTGCTCGTC

Ma03_g13710	AAGGGTGGACACAGATCAAAG	TCAGTGTGTCAACCAAGGAAG
Ma06_g24560	AGCTTCTGGTGCTCAACCTC	GCAGAGGCACTTGTTATGGAG

Table S2. Antibody used in the present study and the antigens.

Antibody	Antigen	Reference
2F4	Un-esterified/Calcium ion cross-linked HG	[1]
CCRC-M34	Partially methyl-esterified HG<br>[base-sensitive homogalacturonan epitope]	[2]
CCRC-M38	Fully de-esterified HG<br>[de-esterified homogalacturonan (DP>5)]	[2]
CCRC-M130	Methyl-esterified on HG	[2]
JIM5	Partially methy-esterified HG epitope: unesterified and partially esterified residues (up to 40%)<br>[Me(alpha)GalA1->4(alpha)GalA1->4(alpha)GalA1->4 (alpha)GalA1->4(alpha)GalA1->4(alpha)MeGalA]	[3]
JIM7	Partially methy-esterified HG epitope: methyl-esterified residues (up to 80%)<br>[GalA1->4MeGalA1->4MeGalA1->4MeGalA1->4MeGalA1->4GalA]	[3,4]
LM7	Partially Me-HG / non-blockwise	[3]
LM18	Lowly methyl-esterified HG / a trigalacturonide	[5]
LM19	De-esterified HG<br>not recognizing oligogalacturonides of DP<4	[5]
LM20	Highly methyl-esterified HG<br>alpha-MeGalA(1-4)alpha-MeGalA(1-4)alpha-MeGalA (1-4)alpha-MeGalA	[5]
PME	Pectin methyl esterase in *Musa acuminata*	Generated by Abmart (Shanghai, China).

DP: degree of polymerization; HG: homogalacturonan; PME: pectin methyl esterase

References

1. Liners F.; Letesson, J.J.; Didembourg, C.; Van-Cutsem, P. Monoclonal Antibodies against Pectin: Recognition of a Conformation Induced by Calcium. *Plant Physiol.* **1989**, *91*, 1419–1424, doi:10.1104/pp.91.4.1419.
2. Pattathil, S.; Avci, U.; Baldwin, D.; Swennes, A.G.; McGill, J.A.; Popper, Z.; Bootten, T.; Albert, A.; Davis, R.H.; Chennareddy, C.; et al. A Comprehensive Toolkit of Plant Cell Wall Glycan-directed Monoclonal Antibodies. **2010**, *153*, 514-525, doi:10.1104/pp.109.151985.
3. Clausen, M.H.; Willats, W.G.T.; Knox, J.P. Synthetic Methyl Hexagalacturonate Hapten Inhibitors of Antihomogalacturonan Monoclonal Antibodies LM7, JIM5 and JIM7. *Carbohydr Res*. **2003**, *338*, 1797-1800, doi:10.1016/S0008-6215(03)00272-6.
4. Knox, J.P.; Linstead, P.J.; King, J.; Cooper, C.; Roberts, K.; Pectin Esterification is Spatially Regulated both within Cell Walls and between Developing Tissues of Root Apices. *Planta* **1990**, *181*, 512-521, doi:10.1007/BF00193004.
5. Verhertbruggen, Y.; Marcus, S.E.; Haeger, A.; Ordaz-Ortiz, J.J.; Knox, J.P. An Extended Set of Monoclonal Antibodies to Pectic Homogalacturonan. *Carbohydr. Res.* **2009**, *344*, 1858–1862, doi: 10.1016/j.carres.2008.11.010.

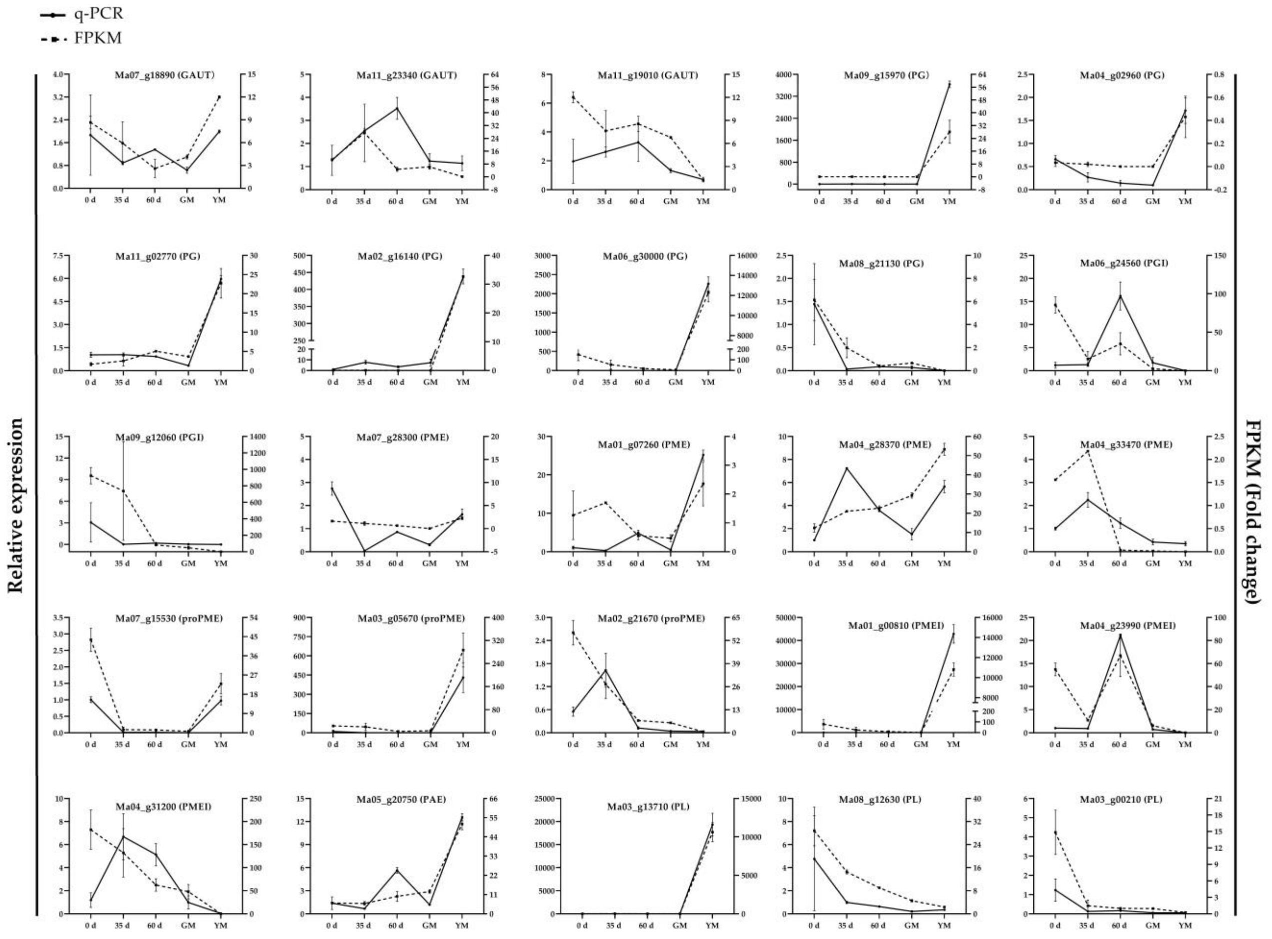

Figure S1. qPCR analysis of the expression of 25 representative differentially expressed homogalacturonan-modifying genes in banana (*Musa* spp. AAA) peel during fruit development and ripening.

0 d: fruits just emerging from the bunch; 35 d: 35 day-old fruits; 60 d: 60 day-old fruits; GM: green matured fruits (at harvest, 85 d); YM: yellow matured fruits (6 d after ethylene treatment).

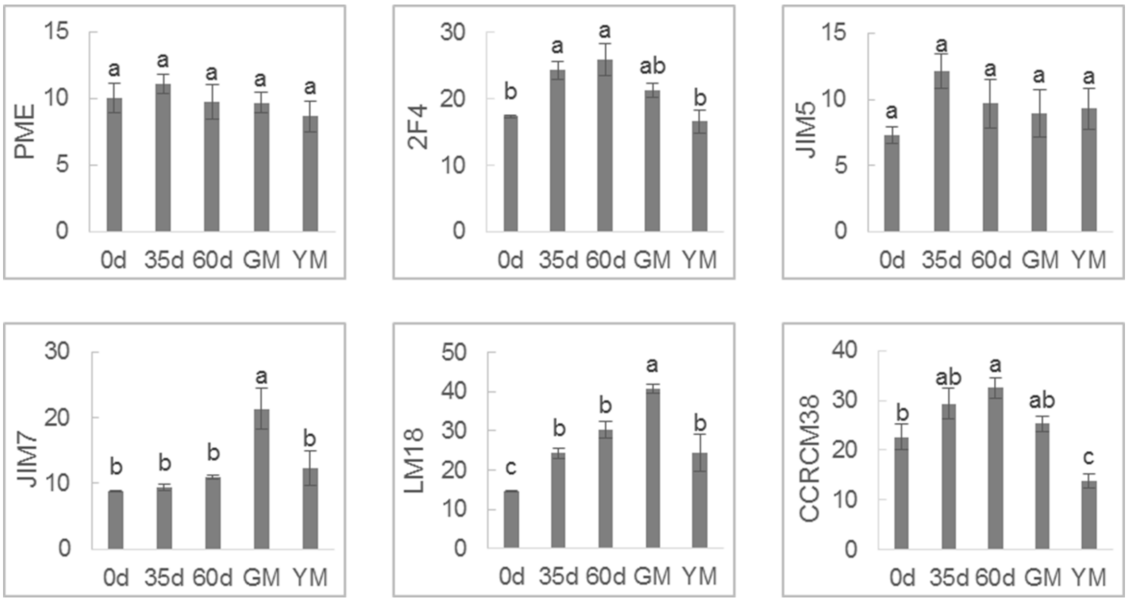

Figure S2. The quantification of fluorescence intensity of antibodies recognizing PME and different homogalacturonan components in banana (*Musa* spp. AAA) peel during fruit development and ripening.

0 d: fruits just emerging from the bunch; 35 d: 35 day-old fruits; 60 d: 60 day-old fruits; GM: green matured fruits (at harvest, 85 d); YM: yellow matured fruits (6 d after ethylene treatment).
